# A Mini-Review on Anion Exchange and Chelating Polymers for Applications in Hydrometallurgy, Environmental Protection, and Biomedicine

**DOI:** 10.3390/polym12040784

**Published:** 2020-04-02

**Authors:** Piotr Cyganowski, Anna Dzimitrowicz

**Affiliations:** 1 Wroclaw University of Science and Technology, Department of Process Engineering and Technology of Polymer and Carbon Materials, Wybrzeze St. Wyspianskiego 27, 50-370 Wroclaw, Poland; 2 Wroclaw University of Science and Technology, Department of Analytical Chemistry and Chemical Metallurgy, Wybrzeze St. Wyspianskiego 27, 50-370 Wroclaw, Poland; anna.dzimitrowicz@pwr.edu.pl

**Keywords:** polymeric nanocomposite, precious metals, resins, cold atmospheric pressure plasma

## Abstract

The rapidly increasing demand for technologies aiming to resolve challenges of separations and environmental protection causes a sharp increase in the demand for ion exchange (IX) and chelating polymers. These unique materials can offer target-selective adsorption properties vital for the removal or recovery of harmful and precious materials, where trace concentrations thereof make other techniques insufficient. Hence, recent achievements in syntheses of IX and chelating resins designed and developed in our research group are discussed within this mini-review. The aim of the present work is to reveal that, due to the diversified and unique physiochemical characteristics of the proposed materials, they are not limited to traditional separation techniques and could be used in multifunctional areas of applications, including catalysis, heat management, and biomedicine.

## 1. Introduction

Ion exchange (IX) and chelating resins are vital for the technological progress of humanity, being a part of separation technologies, recognized as crucial for the development of human society [1]. They are widely used in various processes related to the communal services, as well as a part of modern and advanced technologies that our society attributes progress to. Industrial applications of IX resins include various processes, from preparation of foods (e.g., processing of milk, beverages, and ready-to-use water) to advanced separation technologies (e.g., removal of radioactive isotopes, wastewater treatment, and recovery of precious resources) [2,3,4,5]. Recently, IX resins have drawn even more attraction, becoming a part of end-use industries and households. These emerging areas include softening, demineralization, and purification of drinking water, resulting in locally applied technologies. This covers small-scale plants processing groundwaters for local communities, as well as integrated, compact devices installed for softening of tap water, allowing for the protection of appliances from calcification, and simultaneously reducing a need for cleaning agents. For these reasons, the market of IX resins reveal tremendous potential for further growth, exceeding an estimated worth of 2.0 billion USD by 2027 [6].

Topics related to the IX resins also draw much attention in the scientific communities. The literature provides a number of researches focused on the synthesis, as well as applications [7,8,9,10] of polymers with IX and chelating properties. The research carried out so far provides a vast experimental and literature reviews on conventional and unconventional synthetic routes, modification procedures as well as the nature and properties of functionalities incorporated into IX resins [7,8,9,10,11].

For these reasons, our research group has focused on the development of new polymeric materials with IX and chelating properties designed for the selective separation and recovery of precious metals such as Au, Pt, Pd, and Re. Within the scope of the carried-out research, we have also proposed alternative applications of IX resins, pushing them out of hydrometallurgy. It was done by exploiting the polymeric materials for the synthesis of unique polymeric nanocomposites (pNCs) suitable for multifunctional applications, linking hydrometallurgical purposes with environmental protection engineering and biomedicine.

Hence, the aim of the present work is to summarize recent achievements in the synthesis and applications of IX and chelating resins for separation, catalysis, heat management, and biomedicine developed in our research group. The purpose of the present mini-review is to demonstrate that the polymeric materials with IX and chelating properties are not limited only to the hydrometallurgical and separation processes, but that they can be pushed further beyond. This includes unconventional synthetic routes, as well as applications. 

## 2. Synthetic Protocols for Hydrometallurgy

Traditionally, most of the IX and chelating resins for applications in industrial processes are based on styrene (St) and divinylbenzene (DVB) copolymers (St-co-DVB). Due to the presence of strong covalent bonds, these materials are chemically inactive. Bearing this in mind, it is necessary to introduce chloromethyl groups (–CH_2_Cl) into their structure. The process of such activation is highly undesirable as the alkylating agents, such as bis-chloromethyl ether (BCME), are highly carcinogenic. Consequently, the processes of synthesis require extra measures undertaken to protect the environment, as well as the staff [11,12].

**Figure 1:**
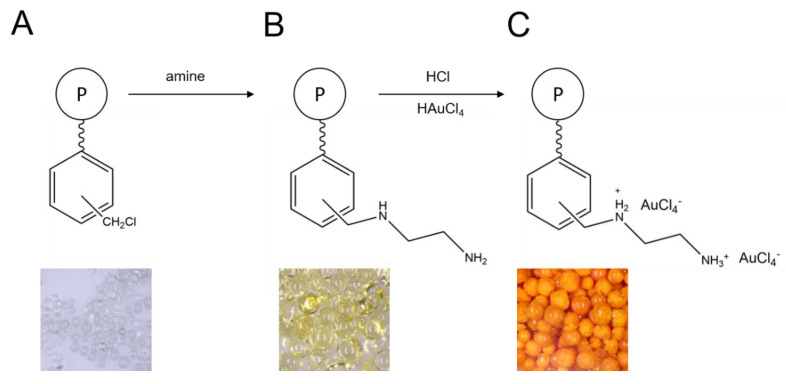
Modification of (**A**) VBC-co-DVB copolymer, using (**B**) an amine (here example for 1,2-diamonoethane), followed by (**C**) an anion exchange (here example for Au(III) ions).

For these reasons, an alternative route involves application of vinylbenzyl chloride (VBC), instead of St, as a functional monomer. Direct copolymerization of VBC with DVB leads to the VBC-co-DVB copolymer production, already possessing –CH_2_Cl groups ready for direct modification. Then, as displayed in Figure 1, the so-obtained copolymer can be further modified to an IX resin by a nucleophilic attack on Cl by, for example, amine or carboxylic acid, resulting in the synthesis of an anion- or cat-ion exchange resin, respectively [11]. As can be seen in Figure 1, the modification process using amines results in the changing of the color of an initial VBC-co-DVB copolymer, from white/transparent to yellow, attributed to the presence of amino-moieties.

Amino functionalities are able to dissociate in solutions of HCl. As a result, the N atom becomes protonated, and Cl^−^ anion is held on an anion exchange resin (see Figure 1C). Another one that functionality reveals an affinity with can easily exchange this excessive anion. This simply defines mechanism of separation of noble metals, as they exist in solutions of electrolytes mainly in anionic forms, such as AuCl_4_^−^, PtCl_6_^−^, PdCl_4_^−^, and ReO_4_^−^ [13,14,15,16]. 

The main drawbacks of applying VBC as a starting monomer for the synthesis of polymeric matrices are related to the reactivity of –CH_2_Cl groups. Conditions of free-radical suspension polymerization (elevated temperature and water-based suspension) make the reactive groups susceptible to hydrolyze, resulting in mers originating from vinyl benzyl alcohol in the formed VBC-co-DVB copolymer or to additionally crosslink thereof through methylene-brides [17]. Both of these undesired side reactions result in decreasing the capacity of a final IX resin, by limiting the number of functionalities or by increasing hydrophobicity of the material, respectively.

Although there are examples exploiting the secondary crosslinking of VBC aimed to increase its affinity toward organic adsorbates, its hydrolysis is highly undesirable, as it simply wastes the possible IX capacity a functional resin can achieve. In the past several years, different factors have been investigated in the view of possible limiting of VBC hydrolysis [18,19]; however, due to the elevated temperatures and prolonged residence of the monomer in water, the issue had not been resolved [20].

To address this problem, our research group decided to take an alternative approach toward the synthesis of VBC-co-DVB copolymers. The research hypothesis was associated with limiting undesired hydrolysis of VBC, which should involve reducing residence of the monomer in water phase first. The influence of the elevated temperature was considered as a secondary factor that could be difficult to avoid, as the benzoyl peroxide (BPO) used as an initiator of polymerization needed 90 °C to generate free radicals. Because of this, microwave (MV) heating was applied for synthesis of starting VBC-co-DVB copolymer. MV heat not only decreases energy consumption, but also enables precise and more volumetric heating, which leads to the significant reduction of time for reactions to be completed [21,22,23,24,25]. The latter one is essential for avoiding hydrolysis of the VBC. 

The procedure of MV-assisted synthesis of VBC-co-DVB copolymer, which based on the standard procedure, involving suspension copolymerization of VBC and DVB monomers in the presence of toluene and in water phase, consisted of poly(vinyl alcohol) and NaCl [26]. The difference was that the duration of the polymerization process was reduced to 4 h, as compared to the conventional method carried out within 25 h [27]. The outcome of the approach exceeded expectations. As displayed in Table 1, despite significantly shortened time of polymerization, MV heating allowed us to achieve 20% greater conversion of monomers (VBC and DVB). Moreover, based on the recorded FT-IR spectra, it was stated that the VBC copolymerized in the MV heat did not hydrolyze. All of that resulted in the fabrication of VBC-co-DVB copolymer with significantly increased concentration of –CH_2_Cl groups (based on Cl concentration) [27], which in further steps improved characteristics of IX resins.

Because MV heat allowed for receiving polymeric matrices with improved characteristics, the undertaken approach was pushed further by carrying out MV-assisted modification of VBC-co-DVB copolymer. Based on the obtained results, it can be stated that utilization of MV heat allows us to reduce energy consumption over 800-times, as compared to the most efficient conventional techniques [28]. Further optimizations allowed for defining optimal reaction environments that modification is carried out in. It was found that, among different organic solvents revealing favorable dielectric constants (defying MV-conductivity), dimethyl formamide (DMF) should not be applied, as the MV field promotes its building-into the VBC-co-DVB copolymer, making the characteristics of IX resins worse. Thus, the reactions should be carried out in dimethyl sulfoxide (DMSO) or N-methyl pyrrolidone (NMP) [29]. Finally, it was possible to link relationships between different reagents, organic solvents, and applied density of MV field [30]. Based on the research carried out, it was possible to conclude that the energy consumption may be even more reduced, as there is no need to apply high input powers and prolonged time of exposure to MVs to receive an IX resin characterized by greatest possible capacity. Even more, when synthesizing anion exchange resins applying amines possessing bis-amino functionality, the power density of MVs should be as low as possible, to avoid additional crosslinking [30]. 

**Figure 2:**
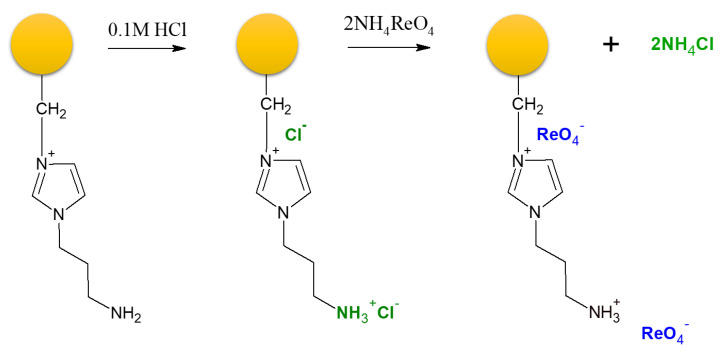
Structure of anion exchange resin obtained in MV heat and simplified mechanism of IX of Re(VII) ions. Figure reproduced from Reference [27], with permission of Elsevier.

Based on these experiences, the “enhanced” VBC-co-DVB copolymer obtained in the MV field [27] was subjected to MV-assisted modification using 1-(3-aminopropyl)imidazole, small power of MV (20 W), and short time (10 min). As displayed in Figure 2, the so-prepared anion exchange resin was then tested toward its adsorption of Re(VII) ions from solutions simulating copper-molybdenum ores. Taking into account the obtained results, the anion exchange resin, based on the enhanced VBC-co-DVB copolymer modified in MV-field, revealed great adsorption capacity (303 mg Re g^−1^), but, most important, the resin revealed a 200-fold greater selectivity toward Re(VII), as compared to excess of Mo(VI), V(V), and Cu(II). This allowed for proposing a solution for the hydrometallurgical challenge of separating Re from Mo [31,32,33,34,35,36]. 

The activity involving new IX and chelating materials for hydrometallurgy was also aimed on the resins for the recovery of Au from wasted electric and electronic equipment (WEEE). Within the carried-out research, we have received a series of *aqua regia* leachates of electronic scrap (CPUs and pin-contact elements), characterized by the trace amount of Au (4 ppm) and over 1000-fold excess of competitive metals (such as Ni, Zn, Cu, etc.). The small concentrations of Au required its quantitative extraction from WEEE to a leachate, hence, it was done using *aqua regia*, as alternatives were much more harmful (cyanides) or much less efficient (thiourea) [37,38,39]. All of this created a serious challenge, as the anion exchange resins designed for such a media should reveal, on the one hand, great selectivity toward Au(III) ions, and on the other hand, they should be resistant to the degradation by *aqua* *regia*.

**Figure 3:**
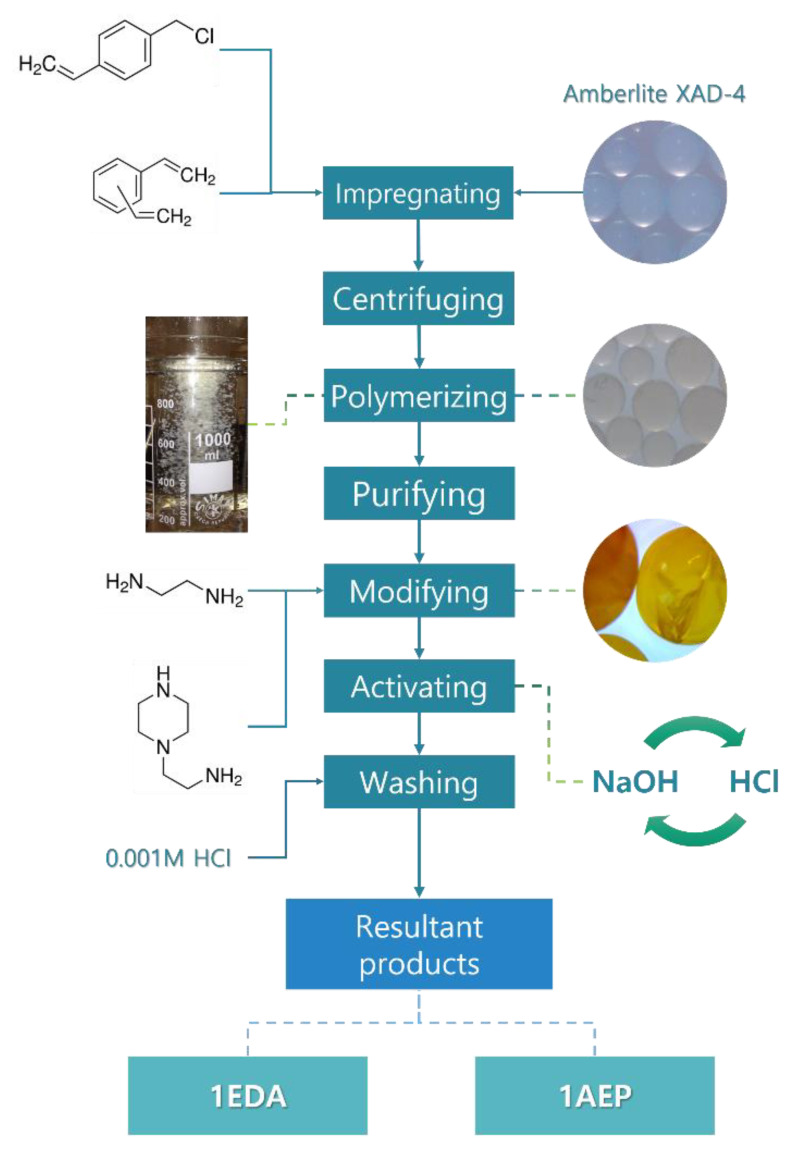
Synthesis of core–shell-like resins form recovery of Au from WEEE. Figure reproduced from Reference [41], with permission of Elsevier.

For this purpose, we developed a new type of amino-functionalized anion exchange resins, revealing a core–shell-like structure. The synthetic protocol, as displayed in Figure 3, involved impregnation of highly crosslinked macroporous Amberlite XAD-4 adsorbent with VBC and DVB monomers further polymerized to create a layer of VBC-co-DVB copolymer functionalized in the next step, using aliphatic and heterocyclic amines. Crosslinked polymeric base (XAD-4) ensured chemical stability of the material, while the localization of the functionalities in its outer layer allowed us to reduce the time of the adsorption process [40]. What is more, selectivity of the resin was ensured by incorporation of 1-(2-aminoethyl)piperazine, revealing a strong affinity toward Au(III) [26,41]. As the result, the anion exchange resin was not only resistant to *aqua regia* but also allowed to receive 100% recovery of Au from WEEE.

The materials synthesized for the selective separation and recovery of Re and Au are set together with others reported in the literature and displayed in Table 2. Based on these, the proposed materials revealed competitive adsorption capacity, selectivity, and durability in the challenging processes of the separation and recovery of the precious metals.

Based on the literature review (see Table 2 for reference), it can be deduced that the proposed anion exchange resins allow us to resolve challenges related to the separation of Re(VII) and Mo(VI), that are particularly important when considering processing Mo-Cu ores, which are the main source of Re. As compared to the literature, the IX resins obtained in MV field led to the omittance of the suppression of ReO_4_^−^ binding ions [34], as well as the necessity of strict control of pH [35], providing at the same time a tempting anion exchange capacity of 303 mg Re g^−^^1^ [27]. 

On the other hand, the proposed core–shell-type IX resins turned out to be resistant to *aqua regia*, allowing us to exclude problematic cyanide leaching of secondary raw materials [37], including possible and effective separation and recovery of Au from WEEE, where Au exists in ultra-trace amounts. Although the literature provides examples of more efficient separation materials [42], it must be noticed that the proposed resins are based on crosslinked suspension copolymers that provide a technological edge as compared to alternative bio-based polymers [42] and enable the use of the most efficient leaching approaches. 

## 3. Nanocomposite Catalysts

### 3.1. In Situ Synthesis Approach

**Figure 4:**
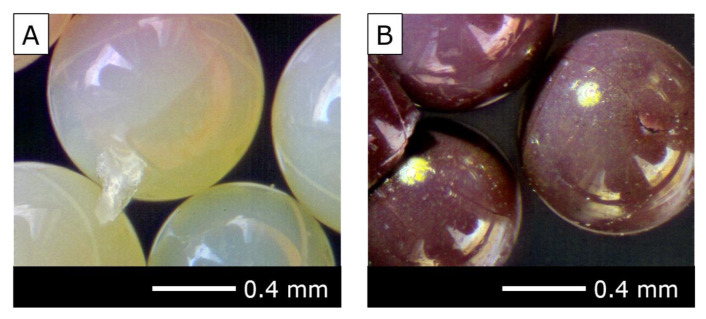
Anion exchange resin (**A**) before and (**B**) after adsorption of Au(III). Figure reproduced from Reference [46], with the permission of Elsevier.

The successful application of core–shell-type anion exchange resins for the recovery of Au from WEEE required a series of optimizations, involving equilibrium studies on adsorption of Au(III), Pt(VI), and Pd(II) from synthetic solutions in 0.1–3 mol L^−1^ HCl. Surprisingly, at lower concentrations of HCl, after adsorption of Au(III), the amino-functionalized resins changed their color to purple instead of the expected orange (see Figure 1C for reference). The phenomenon is displayed in Figure 4.

**Figure 5:**
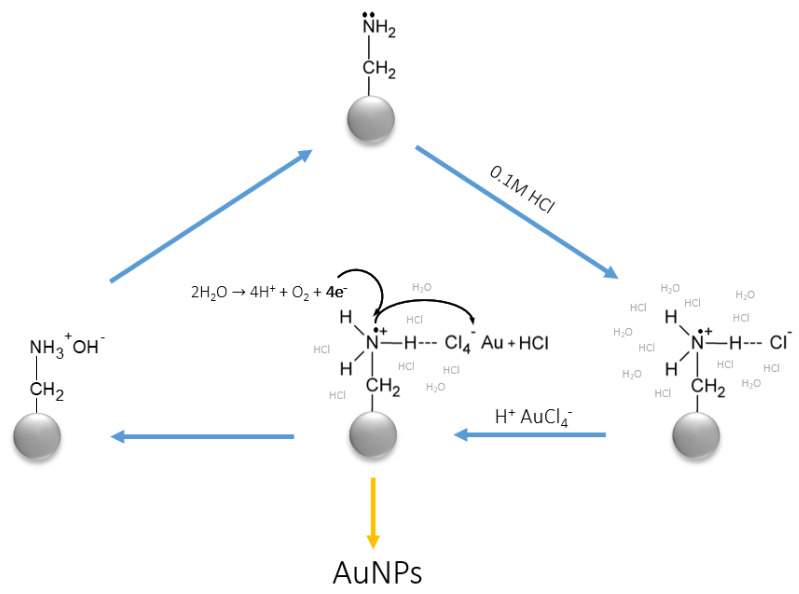
Mechanism of reduction-coupled adsorption of Au(III) on amino-functionalized anion exchange resins. Figure reproduced from Reference [47], with permission of Elsevier.

To explain this phenomenon, it was necessary to take a closer look at the adsorption of Au(III), as well as other noble metals (Pt(VI), Pd(II)) on the synthesized materials. Hence, a series of tests on adsorption capacity and elution of anion exchange resins bearing functionalities derived from 1,2-diamonoethane and 1-(2-aminoethyl)piperazine were tested in the batch tests on the uptake of the noble metals [47]. Based on the detailed equilibrium studies, including analyses performed using Fourier-Transformation Infrared Spectroscopy (FT-IR) and X-Ray Powder Diffractograms (XRD) techniques, the appearance of Au and Pd micro- and nanostructures was not only confirmed but also linked with the changes of chemical structures of the anion exchange resins. A significant observation has been made that the reduction-coupled adsorption of Au(III), Pt(VI), and Pd(II) was followed by the decrease of pH of a solution and generation of oxygen in the mixture, indicating that the noble metals’ ions indeed reduced in the structure of polymers [46,47]. These, linked with the FT-IR spectra of the materials, recorded before and after reduction, allowed us to propose a mechanism that is displayed in Figure 5.

Based on the carried-out research, and a few of the literature reports [48], it was stated that the reduction of Au(III) began after excessive electron located on N atom transferred onto AuCl_4_^−^ chlorocomplex. Then, observed changes of pH and O/O_2_ concentration suggested auto-oxidation of water molecules that generated electrons being able to regenerate amino functionality to reduce next AuCl_4_^−^. The process was very intensive, and the reduction was completed within a few hours, quantitively reducing Au(III). However, because of that, the resultant metallic Au and Pd were characterized by very wide distribution of their particles size, from nano- to micrometers. Moreover, larger particles were precipitated directly into the solution instead of the polymeric matrix, resulting in non-sufficient immobilization. Because reduction was carried out simultaneously with adsorption, it was concluded that the phenomenon was caused by the irregularities in concentration gradient at the solid–liquid interface. At the beginning of the reduction, the concentration gradient was high and decreased with time. As a result, more Au(III) was reduced.

**Figure 6:**
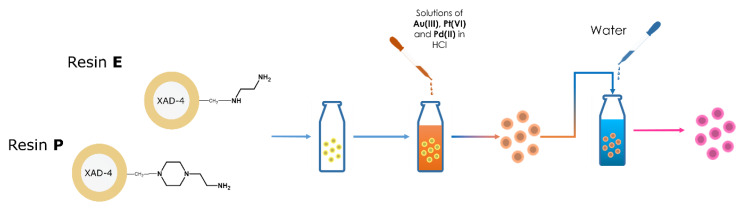
Procedure for preparation of polymeric nanocomposites with noble metals’ nanoparticles. Figure reproduced from Reference [46], with permission from Elsevier.

The solution for synthesizing Au, Pt, and PdNPs of defined granulometric properties was found, knowing the role of H_2_O in the reduction process. As displayed in Figure 6, the adsorption step was carried out in 1 mol L^−1^ HCl that suppressed the reduction [46,49]. Then, the resins saturated with Au(III), Pt(VI), and Pd(II) were transferred to H_2_O, where the reduction began. Because the concentration gradient was defined by the number of moles of adsorbed Au, Pt, and Pd, it was possible to be controlled. 

**Figure 7:**
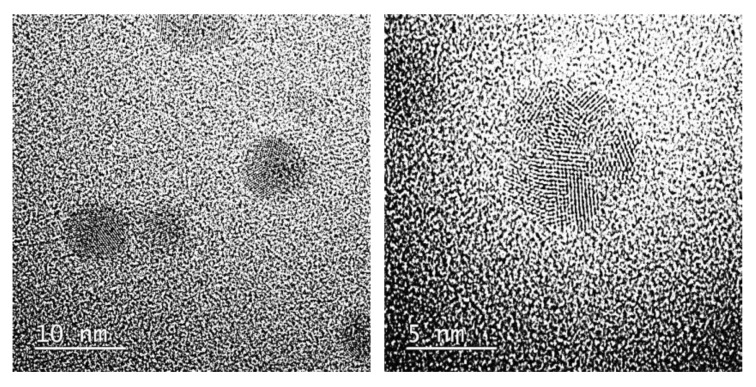
High-Resolution Transmission Electron Microscopy photomicrographs of AuNPs synthesized and stabilized in anion exchange resins. Figure reproduced from Reference [49], with permission from Elsevier.

As it was concluded, the greatest concentration gradient was observed near the surface of a resin’s grain, while the smallest one was deep in the center thereof. Therefore, to obtain smallest in-size NPs, it was necessary to fully saturate a resin first. Then, reduction resulted in the fabrication of AuNPs from 4 to 21 nm in size. The smallest of them are displayed in Figure 7.

Within the carried-out research, a link between an applied amino functionality, acting as a molecular reactor, and the size and shape, as well as concentration, of Au, Pt, and PdNPs was found. Based on the results, it was stated that the smallest in-size NPs can be derived by using anion exchange resins bearing compounds revealing bis-amino functionality (such as 1,2-diaminoethane), while the greatest concentration thereof, was observed by applying amines of a heterocyclic nature [49].

The nanocomposites containing AuNPs [49] or both Au and PdNPs [46] were used as heterogeneous nanocatalysts for the catalytic decomposition of 4-nitrophenol (4-NF) to 4-aminophenol (4-AF). As compared to the other studies [50,51,52,53,54,55,56], the designed catalysts led to the complete decomposition of 4-NF, and what is more, their physical form (suspension copolymers of grain diameter 0.6 mm) allowed their convenient and multiple reuse, without any additional treatment [46,49]. Because within the research project, a second type of nanocatalysts for decomposition of 4-NP was designed, the NCs for applications in catalysis are summarized and compared to the literature further in the text (please refer to Table 4).

### 3.2. Cold Atmospheric Pressure Plasmas Ex Situ Approach

Although the new heterogeneous catalysts turned out to be very efficient, the developed in situ approach requires more investigations on different functional groups and their ability to fabricate NPs of defined granulometric properties. Hence, the ex situ approach, as a more convenient one, was also tested. The ex situ approach allows for the fabrication of NPs outside the polymeric matrix, by applying methods assuring comprehensive control over the optical and granulometric properties thereof. As such, the cold atmospheric pressure plasmas (CAPPs) were applied by our research group.

It is well known that, due to their unique properties, the CAPP-based technologies are widely applied in the industrial and in the know-how sectors. The properties of CAPP-based technologies are associated with the fact that CAPPs are plenteous sources of reactive oxygen species (ROS) and reactive nitrogen species (RNS) [57]. Due to the possibility of controlling the concentration of these ROS and RNS, as well as CAPP operating parameters by constructing and tailoring dedicated for that purpose reaction-discharges systems, it is possible to control the CAPP–liquid interactions of the generated CAPP. For that reason, the CAPP-based technologies find applications in nanotechnology [58], food industry [59], agriculture [60], and biomedicine [61].

**Figure 8:**
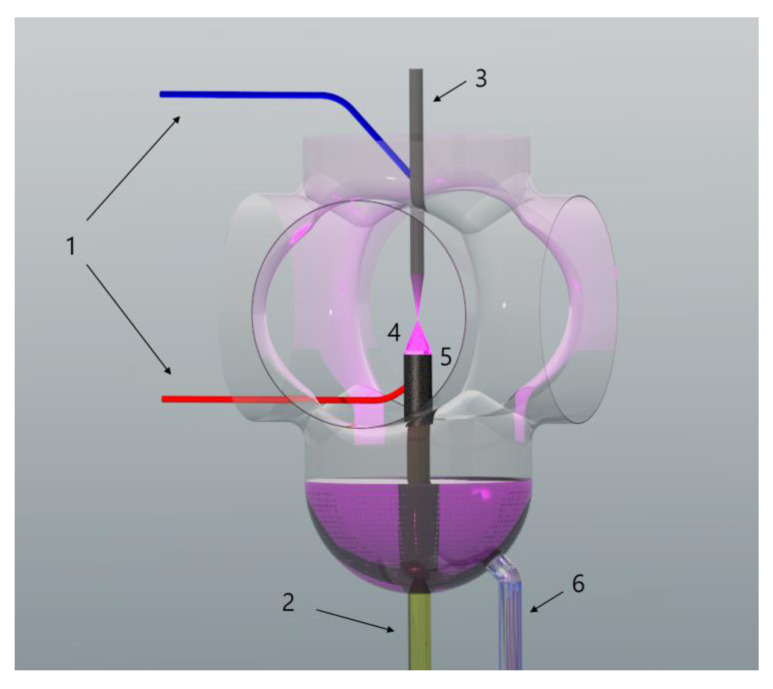
Developed CAPP-based system for continuous production of nanofluids—as an example AuNPs synthesis is shown. (**1**) High-Voltage inputs (+/−); (**2**) a flowing liquid solution, being a precursor to AuNPs; (**3**) tungsten electrode; (**4**) dc-APGD; (**5**) graphite electrode covering quartz capillary; and (**6**) sample reservoir.

A few years ago, a significant increase in the interest was observed for using CAPP-based technologies in nanotechnology due to the fact that the ROS and RNS of defined red-ox properties, generated during the CAPP operation, can be used for the synthesis of nanomaterials [58,62,63,64,65,66,67,68,69,70,71,72]. Bearing in mind a wide range of possibilities for nanomaterials’ applications and simplicity of their CAPP-based synthesis, recently our research group developed an innovative and efficient CAPP-based method for the continuous synthesis of nanomaterials, including AuNPs [73,74,75,76], AgNPs [77,78,79,80,81], Au-Ag@NPs [82], and PtNPs [83]. In contrast to the CAPP-based methods suitable for stationary metallic nanostructures’ synthesis that were developed and applied by other research groups [62,63,64,65,67,68,69,70,71,72], in the proposed method, the metallic nanostructures are being continuously produced in a flow mode (Figure 8).

This highly efficient production of metallic nanostructures is associated with the character of the developed reaction-discharge system, employing as a CAPP source either the direct current atmospheric pressure glow discharge (dc-APGD) [73,74,75,76,77,79,80,81,82,83] or pulse-modulated radio-frequency atmospheric pressure glow discharge (pm-rf-APGD) [78,84]. In our studies, the dc-APGD was generated between either the pin-type tungsten electrode [75,76,77,79,80,81,83] or Ar nozzle [73,74,82] and the surface of flowing liquid electrode, being a NPs’ precursor solution introduced to the designed reaction-discharge systems. On the other hand, the pm-rf-APGD was generated between the pin-type tungsten electrode and the surface of the flowing liquid electrode [78,84]. Since dc-APGD operation led to emit the positive ions or electrons, depending on the polarity of applied electrodes [68], the surface of resulted nanostructures is charged [75], which might exclude the addition of capping agents to the NPs precursor solution [75]. In the case of pm-rf-APGD, the polarity of the electrodes is continuously changed, leading to the alternating production of electrons and positive ions [78]. Typically, the stabilizers are added to this solution in order to preclude the aggregation, agglomeration, and sedimentation of the further obtained nanostructures [74]. Up until now, the synthesized with the aid of CAPP metallic nanostructures were stabilized by using sodium citrate [70], sucrose [71], fructose [64,67,79,85], gelatin [73,74,75,77,82,84], sodium dodecyl sulfate (SDS) [68,69,83], hexadecyltrimethylammonium chloride (CTAC) [62], polyvinyl alcohol [63], poly(vinylpyrrolidone) [65,81], pectins [78,83], and (11-mercaptoundecyl)-N,N,N-trimethylammonium chloride (TMA) alkanethiol [80]. On the other hand, when a stabilizer is not added to the NPs’ precursor solution, the resultant nanostructures might be well dispersed in the solution because of their electrostatic stabilization [73,86].

**Figure 9:**
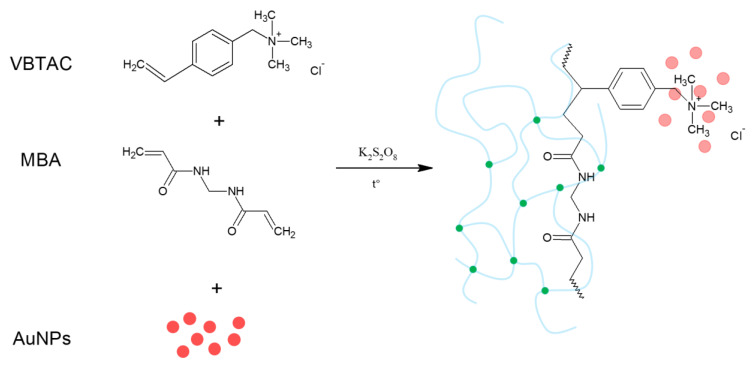
Simplified synthetic route for obtaining hydrogel nanocomposite containing AuNPs. Figure reproduced from Reference [87], with the permission of MDPI, under the Creative Commons Attribution License.

The possibility of the application of CAPP-based technology in the nanomaterials’ synthesis without addition of either reducing or capping agents enables us to produce the uncoated nanomaterials [72,75,76,77,86]. This vital feature led to the possibility of incorporating the obtained nanostructures within the polymeric matrix, in order to produce nanocomposites of defined properties [72,76]. However, such an ex situ approach suffers from diffusional limitations that inorganic NPs immobilized in the polymeric network. Hence, the expected yield of stabilization was expected to be not sufficient. For this reason, the synthesis of NCs with NPs generated in CAPPs was carried out by developing a new *in–ex* situ approach [87]. The uniqueness of this method relies on introducing the suspensions of NPs of defined granulometric properties within a water phase containing hydrophilic monomers, which are further subjected to the free radical polymerization, resulting in the formation of a unique NC, as displayed in Figure 9.

**Figure 10:**
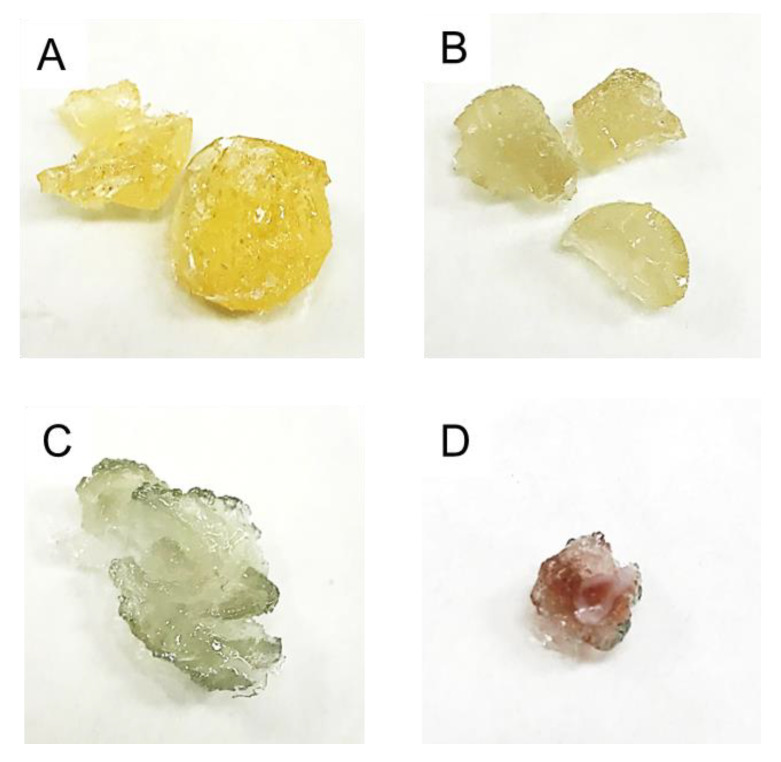
(**A**) VBTAC-co-MBA copolymer; (**B,C**) samples of NCs obtained without NaOH; (**D**) NC with AuNPs after applying NaOH on the VBTAC monomer. Figure reproduced from Reference [87], with the permission of MDPI Creative Commons Attribution License.

The optimization of the procedure was done on the material, resulting from the polymerization-driven immobilization of AuNPs in the vinylbenzyl trimethyl ammonium chloride (VBTAC) and N,N-methylenebis(acryl amide) (MBA), VBTAC-co-MBA copolymer [87]. Although the procedure seemed to be facile, the difficulties that have been met involved the anion-exchange reaction between strong base functionalities of VBTAC and the unreacted AuNPs’ precursor (AuCl_4_^−^). The process resulted in the production of excessive HCl, which digested the AuNPs that should be immobilized within the hydrogel [87]. Hence, the synthesis was altered by forcing the anion-exchange on VBTAC first, using an equivalent amount of NaOH. This approach allowed us to exchange excessive Cl^−^ anion located on VBTAC and neutralize it prior to introducing AuNPs. As displayed in Figure 10, only the sample treated with NaOH prior to the immobilization of AuNPs appeared with the characteristic ruby-red color, and as confirmed, this was successfully synthesized NC [87].

The so-obtained material was tested for its ability to increase heat-exchange rate, in an arrangement simulating cooling systems of modern electronic devices. The AuNP-loaded NC was allowed to increase its cooling rate by 300%, which complies with other reports in the literature that provide similar materials based on polyaniline [88], poly(N-isopropylacrylamide) [89], and stearic acid [90]. The research on NCs for heat-management systems was also tested by using PtNPs produced in the developed-by-us CAPP-based reaction-discharge system, utilizing dc-APGD as a CAPP source. The resultant PtNPs were capped in the poly(vinyl pyrrolidone) that chelating N and O atoms successfully stabilized the NPs, enabling their efficient use as a cooling liquid in an environment simulating cooling systems applied for internal combustion chambers. The heat exchange rate in this case was exceeding water by 800%, as was comparable to commercial NPs-based products, even despite an ultra-trace amount of PtNPs (0.0001%) [81]. As compared in Table 3, the obtained materials turned out to reveal an extraordinary activity, when considering heat exchange rates. The enhancement of heat transfer rates was greater, not only as compared to other nanocomposites, but also to nanofluids being a part of commercial solutions for cooling of internal combustion chambers [27,30], exceeding their positive impact on the heat-management systems. It has been noticed, however, that the price of Pt and Au exceeds by many times the price of Cu- and Al-based systems; however, because the achieved improvement of heat exchange rate was observed for ultra-trace amounts of PtNPs, it can be concluded that the proposed approaches may be a tempting alternative toward the currently used solutions [87]. 

The developed ex situ approach was also used to incorporate raw-AuNPs, prepared with the aid of the dc-APGD into the matrix made of [(2-acryloyloxy)ethyl]trimethylammonium chloride (AOETAC) further crosslinked by applying MBA [76]. The so-obtained material revealed excellent catalytic properties in the controlled reduction of 4-NP to 4-AP [76]. Because within carried-out research projects, we have obtained two types of nanocomposite catalysts—the first type produced by using the in situ approach and the second type produced by using hybrid ex situ protocol—the catalytic activities thereof are set together with other nanocatalysts for decomposition of 4-NP in Table 4.

Based on the literature review, the proposed synthetic routes led to the fabrication of nanocomposite catalysts of favorable catalytic activity. The literature also provides a few examples of nanocatalysts whose activity exceeds the proposed materials [92]; however, it must be remembered that the proposed morphologies (suspension copolymers or hydrogel particles) made the utilization of these nanocatalysts very convenient. Moreover, comparing the catalytic activity of the materials obtained by using the developed approaches with other research in which the dielectric barrier discharge (DBD) was employed to reduce the Ag(I) ions to Ag(0) in order to obtain Ag-TiO_2_ NC [72] for the photocatalytic degradation of methylene blues, as well as in the eradication of the *Escherichia coli* and *Staphylococcus aureus* microorganisms [72], suggests that the NCs synthesized by our research group may reveal extended application beyond catalysis and heat management. In order to extend the applications of the nanostructures obtained by using CAPP technologies in the biomedical field, we have prepared an oil-in-water (O/W) nanoemulsion, consisting of the size-defined raw-AuNPs, produced by using pm-rf-APGD (as a CAPP source), turmeric oil, and gelatin [84]. Basically, the biomedical applications are associated with developing new and useful tools, dedicated to apply them to living beings. For that reason, we have examined the utilization of the O/W nanoemulsion loaded with AuNPs, for breast cancer cell lines migration, and we have assessed its cytotoxicity [84]. Additionally, in the proposed by us O/W nanoemulsion with Au nanostructures, the AuNPs were continuously synthesized in the developed-for-that-purpose reaction-discharge system with pm-rf-APGD. Furthermore, by adjusting the frequency of the pm-rf-APGD, the duty cycle, the concentration of the Au(III) ions in the NPs precursor solution, and the flow rate of the AuNPs precursor solution, it was possible to predict the optimal working parameters under which the defined Au nanostructures would be produced. To reach this aim, the design of the experiment, along with the response surface methodology, was used to find the optimal working parameters of the proposed reaction-discharge system with pm-rf-APGD [84]. By adjusting the frequency to 1600 Hz, the duty cycle to 40%, the concentration of the Au(III) ions in the AuNPs precursor solution to 200 mg L^−1^, and the flow rate of the nanostructures’ precursor solution to 6 mL min^−1^, it was possible to obtain the largest raw-AuNPs of average size of 4.6 ± 1.0 nm (as determined by TEM) [84]. Then, the size-controlled AuNPs were added to the solution consisting of the aqueous solution of gelatin and turmeric oil, forming the O/W nanoemulsion. The so-prepared nanofluid was used in the biomedical field, i.e., in the assessment of the migration and cytotoxic activity against the cancer cells’ lines (human breast cancer cell lines MDA-MB-231 and MCF7 and non-tumor cell line MCF10A) [84]. Comparing our results to nanoemulsion with Au nanostructures produced by others (see Table 5), we found that the method proposed in our research group for nanostructures synthesis allows us to obtain NPs in one step and does not need an additional reducing agent to obtain the AuNPs. The mechanism of this O/W nanoemulsion with AuNPs’ formulation was associated with capping the AuNPs with turmeric oil and gelatin, due to the presence of weak hydrogen bonds between AuNPs, turmeric oil, and gelatin [84]. In addition, the O/W nanoemulsion loaded with Au nanostructures revealed cytotoxicity toward human breast-cancer cell lines MDA-MB-231 and MCF7, which might define the further applications of so-obtained products. 

There are only few examples of similar approaches in the literature. Huang et al. used the O/W nanoemulsion loaded with AuNPs in the inhibition of colon-cancer-cell growth [94]. Other examples, proposed by Fornaguera and Guler, did not provide the applications of the obtained nanoemulsion. There is only presented the putatively uses of the nanoemulsion in biomedical applications [95,96]. This suggests the pioneering nature of the proposed approach.

Based on the obtained results and the literature review, it can be stated that the synthesis and applications of polymer-supported NPs is a promising and tempting alternative toward colloids containing thereof. Although different works provided within this review address the problem of stability and usability of NPs in real-life scenarios, the application of IX and chelating polymers as compared to the literature seems to be particularly interesting, as they offer not only stability of different NPs but also enhanced activity and facile utility thereof. As highlighted within this mini-review, regardless of the form of polymeric base, including crosslinked vinyl- or hydrogel-based, the IX and chelating polymers can reveal successful widespread applications, not limited just to hydrometallurgy, but also including these unconventional. This multifunctionality, linked with ease of use provided by the polymeric matrices, can indeed even progress the nanotechnology toward real-life industrial facilities. 

## 4. Summary

The IX and chelating resins are the types of materials that are mostly recognized by their target-selective separation properties. However, altering the physiochemical structure thereof can lead to a path for synthesis of modern materials, revealing unique properties relevant for catalysis, biomedicine, and beyond. Within the present mini-review, a series of IX and chelating resins revealing such multifunctionality is presented. These materials, possessing amino functionalities (strong- or weak-base), as well as chelating atoms (including O and S), turned out to be very efficient not only in the processes of the recovery of noble and precious metals, but also revealed an ability to synthesize and/or stabilize metallic nanostructures. Such approaches allowed us to obtain a new generation of polymeric nanocomposites, with stable-in-time Au, Pt, and PdNPs characterized by extraordinary performance in heat transfer, catalysis, and biomedicine. 

## 5. Patents

The method for synthesis of core–shell polymeric supports mentioned in this manuscript is protected by Polish Patent no. 225921. The cold atmospheric pressure plasma-based synthesis of metallic nanoparticles is protected by Polish Patent no. 231602.

## Figures and Tables

**Table 1:** Comparison of VBC-co-DVB matrices obtained conventionally and with the aid of the microwave (MV) heat [27].

Copolymer.	Method of Synthesis	Synthesis Time	Conversion of Monomers	Hydrolysis of VBC	Cl ^1^
VBC-co-DVB	Conventional	25 h	70%	+	4.63 ± 0.04
	MV-assisted	4 h	90%	−	5.24 ± 0.04

^1^ Concentration of Cl derived from –CH_2_Cl groups (mmol g^−1^).

**Table 2:** Polymeric adsorbents for the removal of Re and Au [27].

System	Polymeric Base	Active Sites	Re(VII) Uptake	Comments	Ref.
Re + U	Ambersep A920U	Strong base	37 mg g^−1^	Possible Re and U separation	[31]
Re + As + Cu + Fe + Si	ZS70	Weak base	3.15 mg g^−1^	Effective Re separation	[32]
Re	Quaternized poly(4-vinyl)pyridine-co-divinylbenzene		61 mg g^−1^	Up to 170 mg g^−1^ Re(VII) uptake during column studies	[33]
	Reliex 436	Strong base	46 mg g^−1^		
Re + Mo	Purolite A170	Weak base	167 mg g^−1^	Separation of Re and Mo almost impossible.	[34]
Re + Mo	S-co-DVB	4-amino-1,2,4-triazol	354 mg g^−1^	17-fold greater affinity toward Re(VII) when compared to Mo(VI)	[35]
Re + Mo	Merrifield resin	N-methylimidazole	462 mg g^−1^	Resin reveals affinity toward Mo(VI)	[36]
Re + Mo + V + Cu	VBC-co-DVB	1-(3-aminopropyl)imidazole	303 mg g^−1^	Resins obtained applying microwave heat. Affinity toward Re(VII) 200-fold greater when compared to Mo(VI). No adsorption of V and Cu observed	[27] *
			**Au(III) uptake**		
Au iodine–iodide	Purolute A500Plus (S-co-DVB)	Strong base	17 g dm^−3^	Possible 97% of Au recovery from a resin	[43]
Au	Sericin and alginate crosslinked particles		137–648 mg g^−1^	78–84% recovery, using thiourea in HCl	[42]
Au cyanide leachate of PCB	Amberlite IRA400Cl	Strong base	11 mg g^−1^	Up to 100% recovery of Au, using thiourea stripping agent	[44]
Au + Pb	AmberliteXAD7HP		14 mg g^−1^	Reduction-coupled recovery of Au from Pb-based stripping agent	[45]
Au aqua regia leachate of WEEE	Amberlite XAD-4 (S-co-DVB)	1-(2-aminoethyl)piperazine loaded on the core–shell structure	20 mg g^−1^	Resins selective toward trace-quantities of Au, resistant to aqua regia, 100% Au recovery	[41] *

* Works of the authors.

**Table 3:** Enhancement of heat transfer rates, using nanoparticle-based media.

Support	Nanoparticles	Increase of Heat Transfer Rate	Ref.
polyaniline	CuO	12–38%	[88]
poly(N-isopropylacrylamide)	Graphene oxide	~200%	[89]
stearic acid	Carbon nanotubes	91%	[90]
-	Cu, Al_2_O_3_ nanofluid	80%	[27,30]
Poly(vinyl pyrrolidone)	PtNPs	800%	[81] *
(vinylbenzyl)trimethylammonium chloride-co-N,N-methylene bisacrylamide	AuNPs	300%	[87] *

* Works of the authors.

**Table 4:** Nanocomposite AuNPs-based catalysts for catalytic decomposition of 4-NP [49].

Support	Average Size of AuNPs (nm)	*k_m_* (mg^−1^ min^−1^)	Ref.
Aryl diazonium-modified olive waste	n.a.	0.02	[51]
PMMA	7.0	0.37	[52]
Magnetite microspheres	5.0–7.0	1.22	[53]
Reductive carbon dots	15.0	0.10	[54]
ZrO_2_	17.0 ± 5.1	0.06	[56]
Egg-shell	6.2 ± 4.0	1.07	[91]
Fe_3_O_4_	15.0	13.8	[92]
Sulfur doped graphitic carbon nitride	12.0	1.50	[93]
Quaternary ammonium hydrogel	6.2 ± 1.2	0.10	[76] *
VBC/DVB with 1-amino-4-methylpiperazine ligands	4.5 ± 2.1	1.46	[49] *

* Works of the authors.

**Table 5:** The selected examples of the nanoemulsions applications in the biomedical field.

Components of Reaction Mixture	Average Size of AuNPs (nm)	Applications	Ref.
AuNPs, Polysorbate 80, PLGA	15	Biomedical, not defined	[95]
AuNPs, *Calendula officinalis*, Tween 85, *Nigella sativa* oil, lecithin	40	Bioactive agent	[96]
AuNPs, Tween 80, Lycopene	21.3 ± 3.7	Colon cancer cells	[94]
AuNPs, Gelatin, Turmeric oil	4.6 ± 1.0	Breast cancer cells	[84]
